# Supplementary material for: (p)ppGpp-mediated GTP homeostasis ensures survival and antibiotic tolerance of Staphylococcus aureus
Source: Commun Biol. 2025 Mar 28;8:508. doi: 10.1038/s42003-025-07910-6 (PMC11953324; doi:10.1038/s42003-025-07910-6)
Supplement: Supplementary file 2 — Supplementary information [file 42003_2025_7910_MOESM2_ESM.pdf]

(p)ppGpp-mediated GTP homeostasis ensures survival and antibiotic tolerance of Staphylococcus aureus.

Salzer et al.,

Supplemental tables and figures

Table S1 Strains

**Strain name**	**Description**	**Reference**
Escherichia coli		
IM08B	SA08BΩP_{N25}-*hsdS* (CC8-1) (SAUSA300_0406) of NRS384 integrated between the *essQ* and *cspB* genes	1
Staphylococcus aureus		
HG001 WT	wildtype, RN1 derivate, *rsbU* repaired	2,3
CYl316	RN4220(pYL112Δ19), L54 int gene,	4
HG001 (p)ppGpp^0	HG001-229-230-263, Mutation in the synthetase domain of *relP* ,*relQ* and complete deletion of *rel* (Δ$relP_{syn}$ Δ$relQ_{syn}$ Δrel)	5
USA300 JE2 WT	USA300 derivative, cured of all plasmids	NARSA
USA300 JE2 (p)ppGpp^0	USA300 JE2 -229-230-263, Mutation in the synthetase domain of *relP* ,*relQ* and complete deletion of *rel* (Δ$relP_{syn}$ Δ$relQ_{syn}$ Δrel)	6
HG001 (p)ppGpp^0 rsh_{syn} compl.	HG001-229-230-263 pCG199	This work
HG001 (p)ppGpp^0 *relP* compl.		7
HG001 (p)ppGpp^0 *relQ* compl.		7
HG001 *ΔcodY*	HG001-21, *ΔcodY::tetM*	2
HG001 (p)ppGpp^0 *ΔcodY*		7
HG001 *ΔpurR*	NE1237, T*nbursa::purR, erm*^{R}	This work
HG001 *ΔguaBA*	HG001-337, markerless *ΔguaBA* mutation	8
HG001 (p)ppGpp^0 *ΔguaBA*	HG001-229-230-263-337	8
HG001 *ΔguaBA* compl.	HG001-337, pCG115	This work
HG001 (p)ppGpp^0 *ΔguaBA* compl.	HG001-229-230-263-337, pCG115	This work
HG001 WT-PqoxABCDmut	TSS +1 of P*qoxABCD* is mutated from A → G	This work
HG001 (p)ppGpp^0-PqoxABCDmut	TSS +1 of P*qoxABCD* is mutated from A → G	This work
SH1000 WT	Phage-cured, and *rsbU* repaired derivative of RN1	9
SH1000 (p)ppGpp^0	SH1000 -229-230-263, Mutation in the synthetase domain of *relP* ,*relQ* and complete deletion of *rel* (Δ$relP_{syn}$ Δ$relQ_{syn}$ Δrel)	This work

Table S2 Plasmids

Plasmid	Desription	Reference
pIMAY-Z	Carries Gram-positive ribosome binding site and *lacZ* cloned downstream from the constitutive *cat* gene in pIMAY; 8.8 kb, Cm^r	1
pCG3	Integration vector, integrates into *geh*, *erm*	10
pCG199	Integrative rsh_{syn} complementation plasmid, pCL84-based	11
pCG216	Integrative *relQ* complementation plasmid	7
pCG833	Integrative *relP* complementation plasmid	7
pCG919	pIMAY-Z based plasmid for mutation of TSS+1 P*qoxABCD* is mutated from A → G	This work
pCG229	pKOR1 with integrated, mutated *relP*	5
pCG230	pKOR1 with integrated, mutated *relQ*	5
pCG263	pKOR1 with integrated, mutated *rel*	5
pCG115	integration vector for complementation, *xpt, pbuX, guaB, guaA, erm*	This work

Table S3 Oligonucleotides

Primer name	Sequence 5‘ → 3‘	Purpose
**psm2391**	CATCGTTTTGTCCTCCTG	qRT-PCR *psmα*
**psm271**	TCATCGCTGGCATCATTA	
**rpslfor**	ACCACAAAAACGTGGTGTATGTACT	qRT-PCR *rpsL*
**rpslseqrev**	ACCAGGGATGTATGCGTT	
**rsaD-LCfor**	GGTAATACACTTGGCTTTTATGGG	qRT-PCR *rsaD*
**rsaD-LCrev**	AGAAGTTATCTCCTTTGTGTTG	
**qoxA-LCfor**	TCTTTGCTTCTATTATTTGGC	qRT-PCR *qoxA*
**qoxA-LCrev**	GCATGAAGACGATTGAATAAAG	
**gyr297**	TTAGTGTGGGAAATTGTCGATAAT	qRT-PCR *gyrB*
**gyr574**	AGTCTTGTGACAATGCGTTTACA	
**pCG919gibfor**	AATTCCTGCAGCCCGGGGTTGTTAT ATGGTTCGTCATTTCCA	Insert 1 for cloning of pCG919mut
**pCG919-2mutfor**	CTTAAAATTAATGTTGAGCCCTACAT TTGTAG	
**pCG919-2mutrev**	GTAGGGCTCAACATTAATTTTAAGTT ATTACAC	Insert 2 for cloning of pCG919mut
**pCG919gibrev**	GCCGCTCTAGAACTAGTGTCTAGTC AGGGGCCCCAAC	
**pCG919seqfor1**	TTGTTATTCTAACTTCATCTGCAAC	Sequencing primer
**Pqox-ctrlfor**	GAACCCACGTCACACCTTGA	
**relPDIG-for**	GTCGCACATTCTTTCAG	verification of *relP* synthase mutant
**relPDIG-rev**	CGTTATTAGGTTTCGTAGAGTT	
**relQDIGfor2**	TTCGTAACACTAAAGAAAGTGG	Verification of *relQ* synthase mutant
**relQDIGrev2**	GCGTGTAATATTTTTGAGCT	
**rel431for**	GCGTGGCTTTATCATTGG	Verifikation of rel_{syn} mutant
**relLC4rev**	ACTTCAACCATCATTCGG	
**scv1**	GCAACACCACATAATGGTTCA	Verification of integration of pCG115
**pCG3intrev**	ACATAGCGTTGCCTTGGTAG	
**scv2.1**	TGTGCCATGATAACAGCACG	Verification of integration of pCG115
**pCG3intfor**	GCCATACCACAGATGTTCCA	
**BamguaAc-for2**	CCCCGGATCCATAGTTGTCGCCCCT TTAAAAA	pCG115, gua comp.
**BamguaAc-rev**	CCCCGGATCCAATTAAAACGCAAAC TTAGAACA	pCG115, gua comp.

Gating strategy for flow cytometry (FACs) plots

Bacterial gating occurred at the FSC/SCC density plot omitting PBS-derived signals. Mean bacteria per gate 19769,875 (± SD 128) representing 96,75 % (± SD 1,36) of the population (Fig. S7A).

Supplemental figures Salzer et al.,

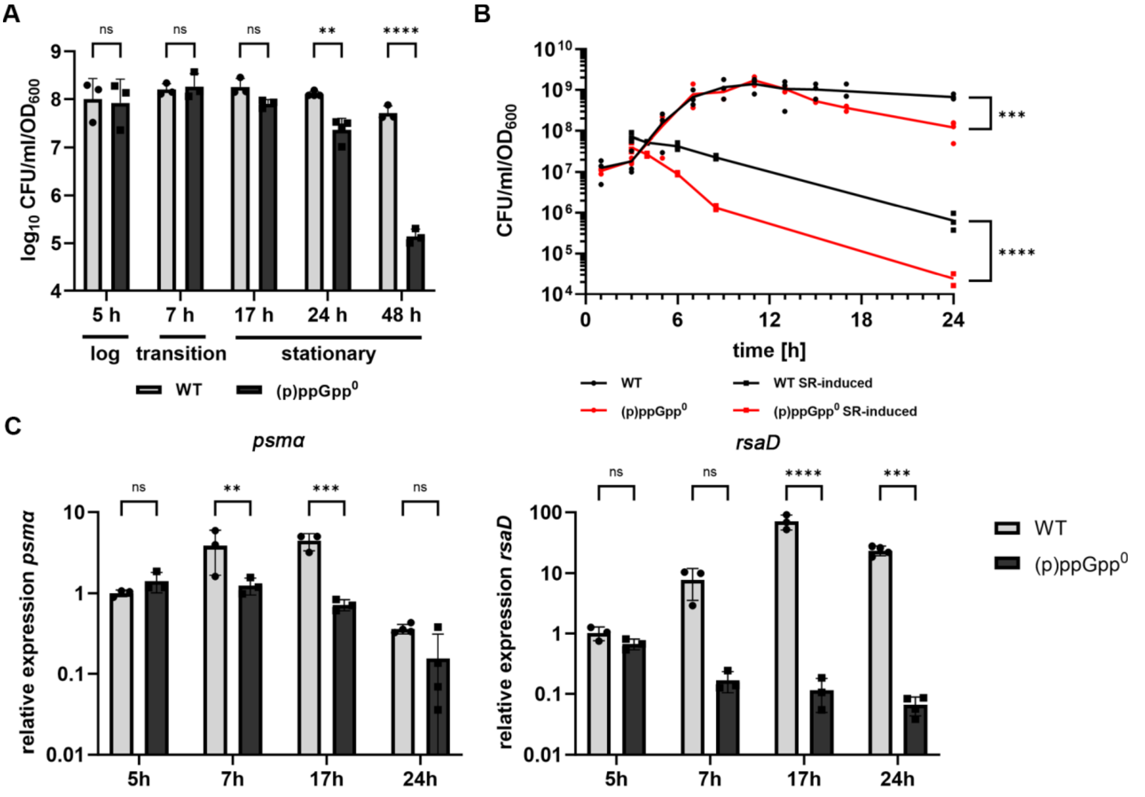

Fig. S1 Decreasing culturability of (p)ppGpp^0 cells during stationary phase and stringent response induction

(A) Culturability of HG001 wildtype (WT) and (p)ppGpp^0 mutant was evaluated during different growth stages by CFU/ml enumeration normalized to OD_{600}. Data shown are mean of ± SD (n=3 biological replicates). Statistical significance was determined by a two-way analysis of variance (ANOVA) with Šidák's post-test performed on $\log_{10}$ transformed data (**p-value =0.0018, (****p-value <0.0001, ns p-value =0.997 for 5 h, 0.999 for 7 h, =0.04213 for 17 h). (B) Bacteria grown to exponential growth phase were treated with sub-inhibitory concentrations of mupirocin (0,125 µg/ml) (stringent response (SR)-induced) and culturability was determined by CFU/ml enumeration normalized to OD_{600} (n=3 biological replicates). For comparision untreated strains are included. Statistical significance was determined by a two-way analysis of variance (ANOVA) with Tukey`s multicomparion post-test performed on log10 transformed data (***p-value =0.0009, ****p-value <0.0001) (C, D) Bacterial cells were harvested during different growth phases and total RNA was isolated. *psmα and rsaD* transcript levels were

determined by RT-qPCR and normalized to *gyrB* expression by ΔΔCt method. Data are shown as mean ± SD (n=3 biological replicates). Statistical significance was determined by two-way analysis of variance (ANOVA) with Šidák's post-test (C: **p-value =0.0045, ***p-value =0.0001, ns p-value = 0.957 for 5 h, 0.9943 for 24 h); (D: (****p-value <0.0001, ***p-value =0.0005, ns p-value =0.999 for 6h, 0.5764 for 7 h).

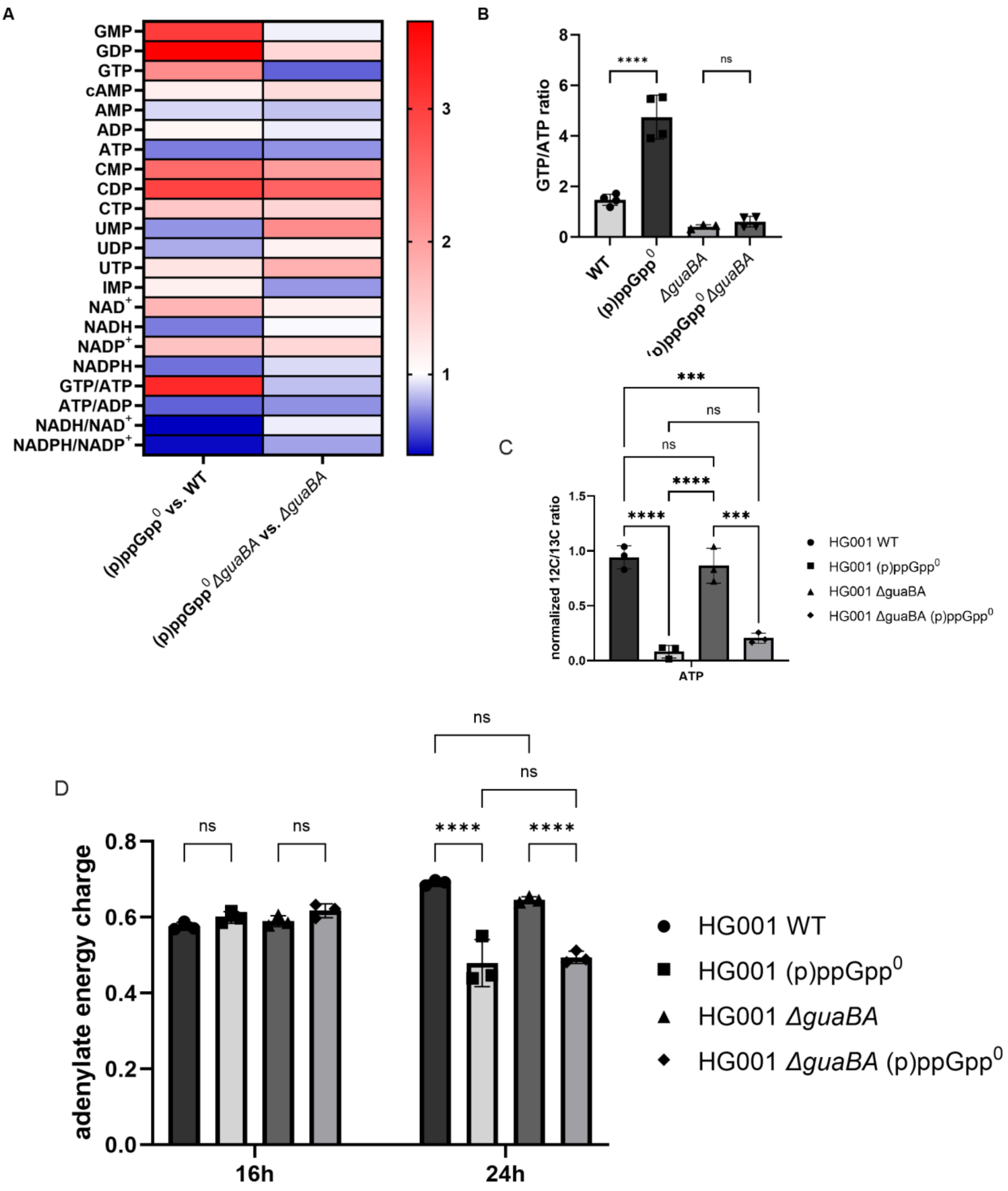

Fig. S2 Determination of nucleotide levels in late stationary phase cells.

(A) Metabolites were extracted and nucleotides were measured and quantified via LC-MS/MS from cells grown to late stationary growth phase (24h). Shwon are relative nucleotide levels as determined by comparing (p)ppGpp^0 vs. WT and (p)ppGpp^0 *ΔguaBA* vs. *ΔguaBA*. (B) The GTP/ATP ratio. Data shown are mean of ± SD (n=4). Statistical significance was determined by one-way analysis of variance (ANOVA) with Tukey's post-test (****p-value <0.0001, ns p-value =0.128). (C) ATP ratio. Data shown are mean of ± SD (n≥4 biological replicates). Statistical significance was determined by one-way analysis of variance (ANOVA) with Tukey's post-test (****p-value <0.0001, ***p-value =0.0002, ns p-value =0.8022 for guaBA versus WT, 0.4921 for guaBA/(p)ppGpp^0 versus (p)ppGpp^0). (E) the adenylate energy charge were calculated from the relative nucleotide level [ATP] + 0.5 [ADP]/[ATP] + [ADP] + [AMP]. Statistical significance was determined by one-way analysis of variance (ANOVA) with Tukey's post-test (****p-value <0.0001, ns p-value =0.7423 (p)ppGpp^0 versus WT, 16h, 0.624 for *guaBA*/(p)ppGpp^0 versus *guaBA*, 16 h, 0.1857 for *guaBA* versus WT 24 h, 0.9285 *guaBA*/pppGpp^0 versus (p)ppGpp^0 24 h).

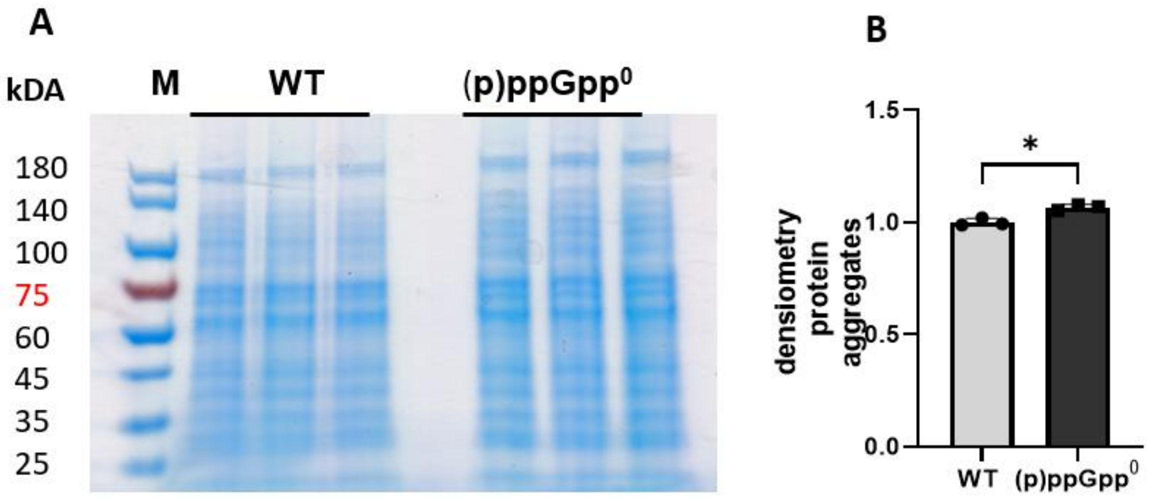

Fig. S3 Quantification of protein aggreagates.

(A) Protein aggregates were isolated from wildtype and (p)ppGpp^0 cells grown to late stationary phase and anaylsed by SDS-PAGE and Coomassie staining. M: protein marker. For each strain, protein aggregates were isolated from three individual biological replicates (n=3). (B) Protein aggregates were quantified by densiometric analysis. Data shown are mean of ± SD (n=6 biological replicates from two individual experiments). Statistical significance was determined by a two-tailed, unpaired t-test (**p-value =0.0066).

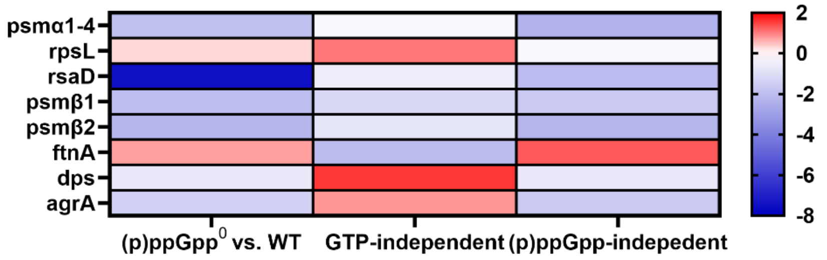

Fig. S4 RNA-seq analysis of stringent response genes

Heatmaps displaying the RNA-seq fold change of selected stringent response regulated genes. Log_2 fold changes in relative transcript abundances are colour-coded with red and blue, indicating up- and downregulation, respectively. “GTP-independent” compares the expression levels of (p)ppGpp^0 *ΔguaBA* vs. *ΔguaBA,* while “(p)ppGpp-independent” compares the expression levels of (p)ppGpp^0 vs. (p)ppGpp^0 *ΔguaBA*.

The RNA-seq data shown are from three individual biological replicates (n=3). Full RNA-seq data are available in Table S1 and S2 in the supplementary material.

Fig. S5 Antibiotic tolerance in exponential and stationary phase.

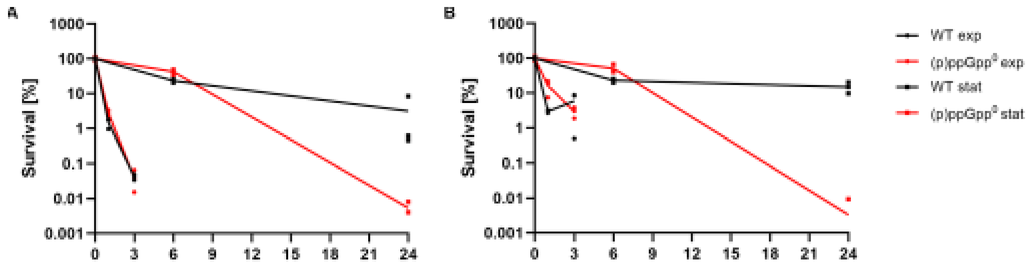

(A) Stationary phase (■) or mid-exponential phase (●) bacteria were treated with 100x MIC ciprofloxacin (A) or oxacillin (B) and survival was calculated in comparison to untreated cells at different time points after treatment (n=3 biological replicates).

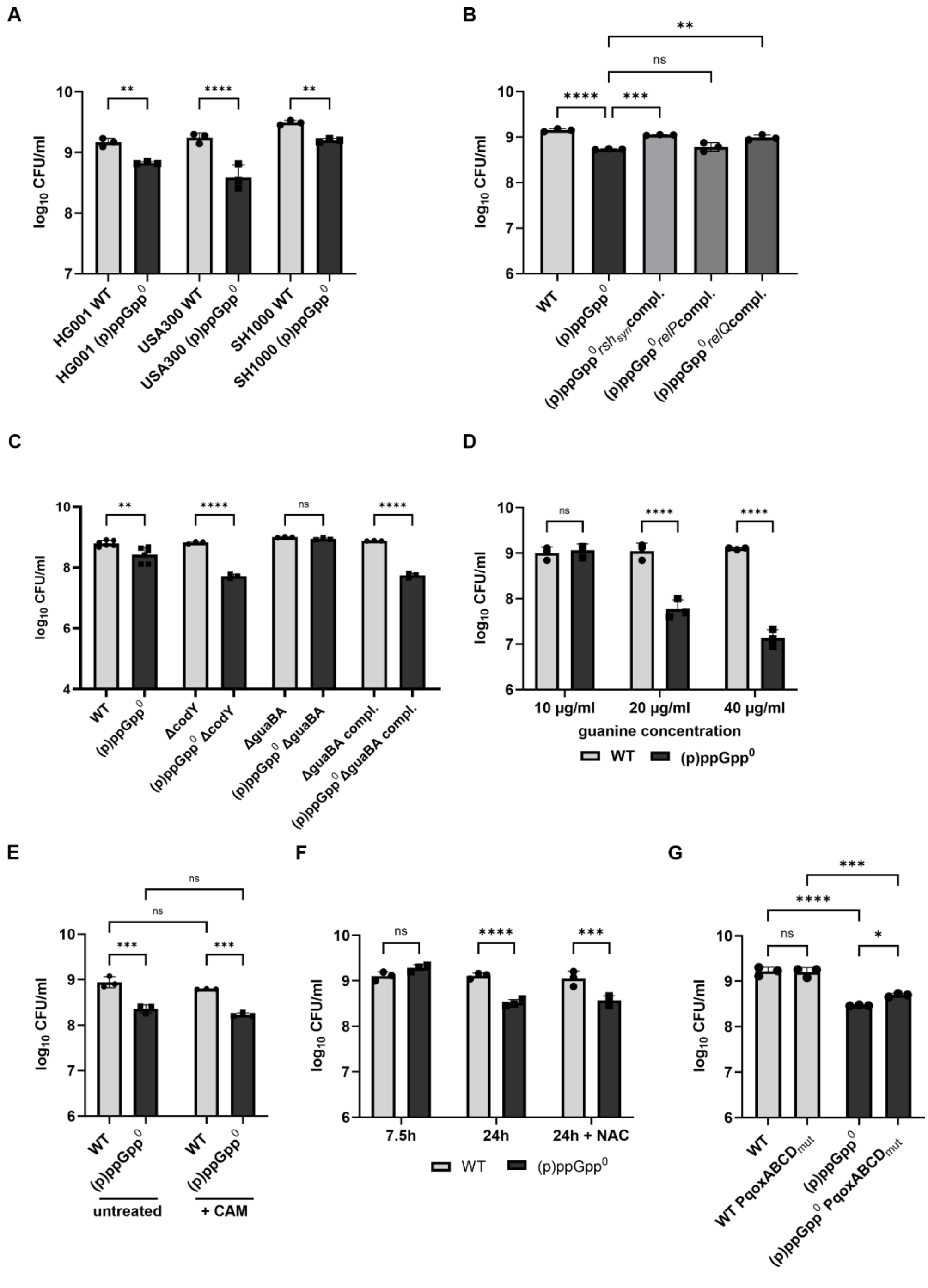

Fig. S6 Culturability determined by CFU/ml.

(A - E) Strains were grown for 24h in CDM and culturability was determined by CFU/ml enumeration [compare to Fig. 1D&E, Fig. 2B&C, Fig 4C, Fig 5B]. Data shown are mean ± SD (n ≥ 3 biological replicates). Statistical significance was determined by one-way analysis of variance (ANOVA) with a Šidák's post-test on $\log_{10}$ transformed data (A: ****p-value <0.0001, **p-value = 0.0095, ns p-value = 0.030), (B: ****p-value <0.0001, ***p-value =0002, **p-value =0013, ns p-value = 0.975), (C: ****p-value <0.0001, **p-value =0016, ns p-value =0.9983), (D: ****p-value <0.0001, ns p-value=0.945), (E: ***p-value

=0.0001, ns p-value = 0.999 for WT untreated versus CAM, 0.997 for pppGpp untreated versus CAM), (F: : ****p-value <0.0001, ***p-value =0.0002, ns p-value =0.1425).

A

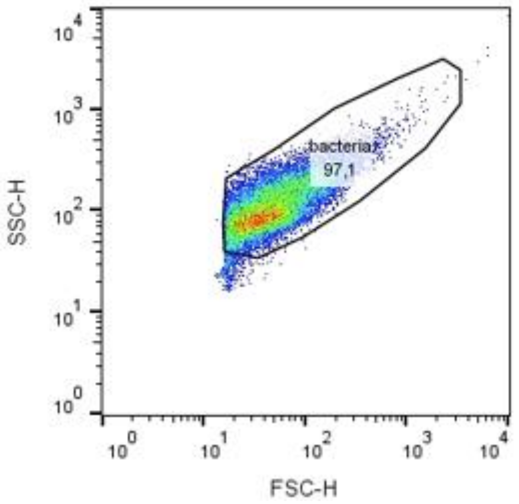

B

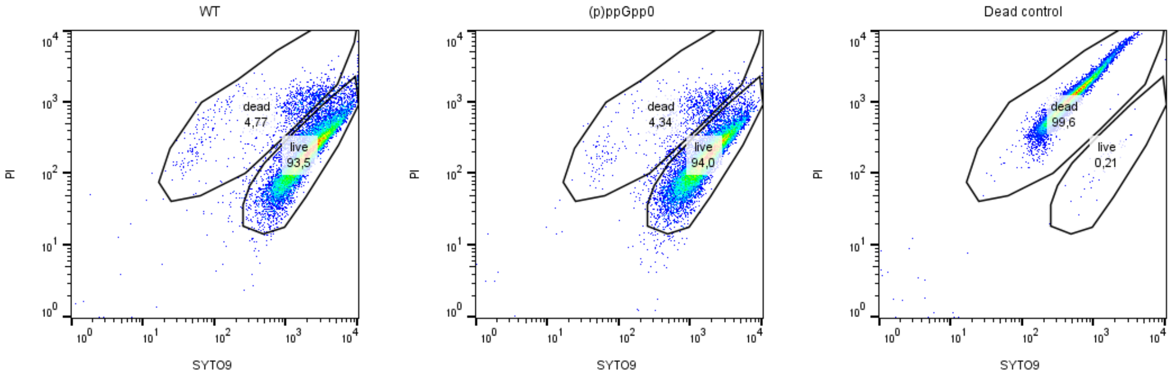

Fig. S7 LIVE/DEAD assessment using flow cytometry

Bacterial cultures were washed in 0.85% NaCl prior to staining for bacterial viability, as recommended by the manufacturer (Live/Dead BacLight^{TM} Bacterial Viability Kit). All cultures were further diluted to an OD_{600} of 0.2-0.4 before staining with the Syto 9 and propidium iodide dye mixture (1:1 dye ratio). 50 µl of stained bacteria were mixed with 200 µl 0.85% NaCl and analysed in the FACSCalibur flow cytometer (Becton Dickinson) using the CellQuest Pro software (Becton Dickinson). **(A)** Bacterial gating occurred at the FSC/SCC density plot omitting PBS-derived signals. Mean bacteria per gate 19769,875 (± SD 128) representing 96,75 % (± SD 1,36) of the population. (**B)** Representative results of live/dead ratio for WT, pppGpp^{0} mutant and dead WT control (treated with 70% ethanol, 15 min) using FL1 (530/30 nm, green) and FL3 (>670 nm red) fluorescence detectors.

Supplementary References

1 Monk, I. R., Tree, J. J., Howden, B. P., Stinear, T. P. & Foster, T. J. Complete Bypass of Restriction Systems for Major Staphylococcus aureus Lineages. mBio 6, e00308-00315 (2015). https://doi.org/10.1128/mBio.00308-15

2 Pohl, K. et al. CodY in Staphylococcus aureus: a regulatory link between metabolism and virulence gene expression. Journal of bacteriology 191, 2953-2963 (2009). https://doi.org/10.1128/JB.01492-08

3 Herbert, S. et al. Repair of global regulators in Staphylococcus aureus 8325 and comparative analysis with other clinical isolates. Infection and immunity 78, 2877-2889 (2010). https://doi.org/IAI.00088-10 [pii] 10.1128/IAI.00088-10

4 Lee, C. Y., Buranen, S. L. & Ye, Z. H. Construction of single-copy integration vectors for Staphylococcus aureus. Gene 103, 101-105 (1991).

5 Geiger, T., Kastle, B., Gratani, F. L., Goerke, C. & Wolz, C. Two small (p)ppGpp synthases in Staphylococcus aureus mediate tolerance against cell envelope stress conditions. Journal of bacteriology 196, 894-902 (2014). https://doi.org/10.1128/JB.01201-13

6 Horvatek, P. et al. Inducible expression of (pp)pGpp synthetases in Staphylococcus aureus is associated with activation of stress response genes. PLoS genetics 16, e1009282 (2020). https://doi.org/10.1371/journal.pgen.1009282

7 Salzer, A., Keinhörster, D., Kästle, C., Kästle, B. & Wolz, C. Small Alarmone Synthetases RelP and RelQ of Staphylococcus aureus Are Involved in Biofilm Formation and Maintenance Under Cell Wall Stress Conditions. Frontiers in microbiology 11, 575882 (2020). https://doi.org/10.3389/fmicb.2020.575882

8 Kastle, B. et al. rRNA regulation during growth and under stringent conditions in Staphylococcus aureus. Environmental microbiology 17, 4394-4405 (2015). https://doi.org/10.1111/1462-2920.12867

9 Horsburgh, M. J. et al. sigmaB modulates virulence determinant expression and stress resistance: characterization of a functional rsbU strain derived from Staphylococcus aureus 8325-4. Journal of bacteriology 184, 5457-5467 (2002). https://doi.org/10.1128/jb.184.19.5457-5467.2002

10 Mainiero, M. et al. Differential target gene activation by the Staphylococcus aureus two-component system saeRS. Journal of bacteriology 192, 613-623 (2010). https://doi.org/10.1128/JB.01242-09

11 Geiger, T. et al. Role of the (p)ppGpp synthase RSH, a RelA/SpoT homolog, in stringent response and virulence of Staphylococcus aureus. Infection and immunity 78, 1873-1883 (2010). https://doi.org/10.1128/IAI.01439-09
