# Supplementary material for: (p)ppGpp-mediated GTP homeostasis ensures survival and antibiotic tolerance of Staphylococcus aureus
Source: Commun Biol. 2025 Mar 28;8:508. doi: 10.1038/s42003-025-07910-6 (PMC11953324; doi:10.1038/s42003-025-07910-6)
Supplement: Supplementary file 3 — Description of Additional Supplementary File [file 42003_2025_7910_MOESM3_ESM.pdf]

Description Of Additional Supplementary File

File name: Supplementary Data 1
Description: RNA seq results raw data

File name: Supplementary Data 2
Description: RNA seq results significant genes only

File name: Supplementary Data 3
Description: Metabolomics Data

File name: Supplementary Data 4
Description: The source data behind the graphs in the paper
